# Supplementary material for: The interactome of intact mitochondria by cross-linking mass spectrometry provides evidence for coexisting respiratory supercomplexes
Source: Mol Cell Proteomics. 2017 Dec 8;17(2):216–32. doi: 10.1074/mcp.RA117.000470 (PMC5795388; doi:10.1074/mcp.RA117.000470)
Supplement: Supplemental Data [file supp_RA117.000470_133922_0_supp_23978_fzffwf.zip › spectra_annotation/mito_DR_spectra_annotation/184-1-4-1-3-1.pdf]

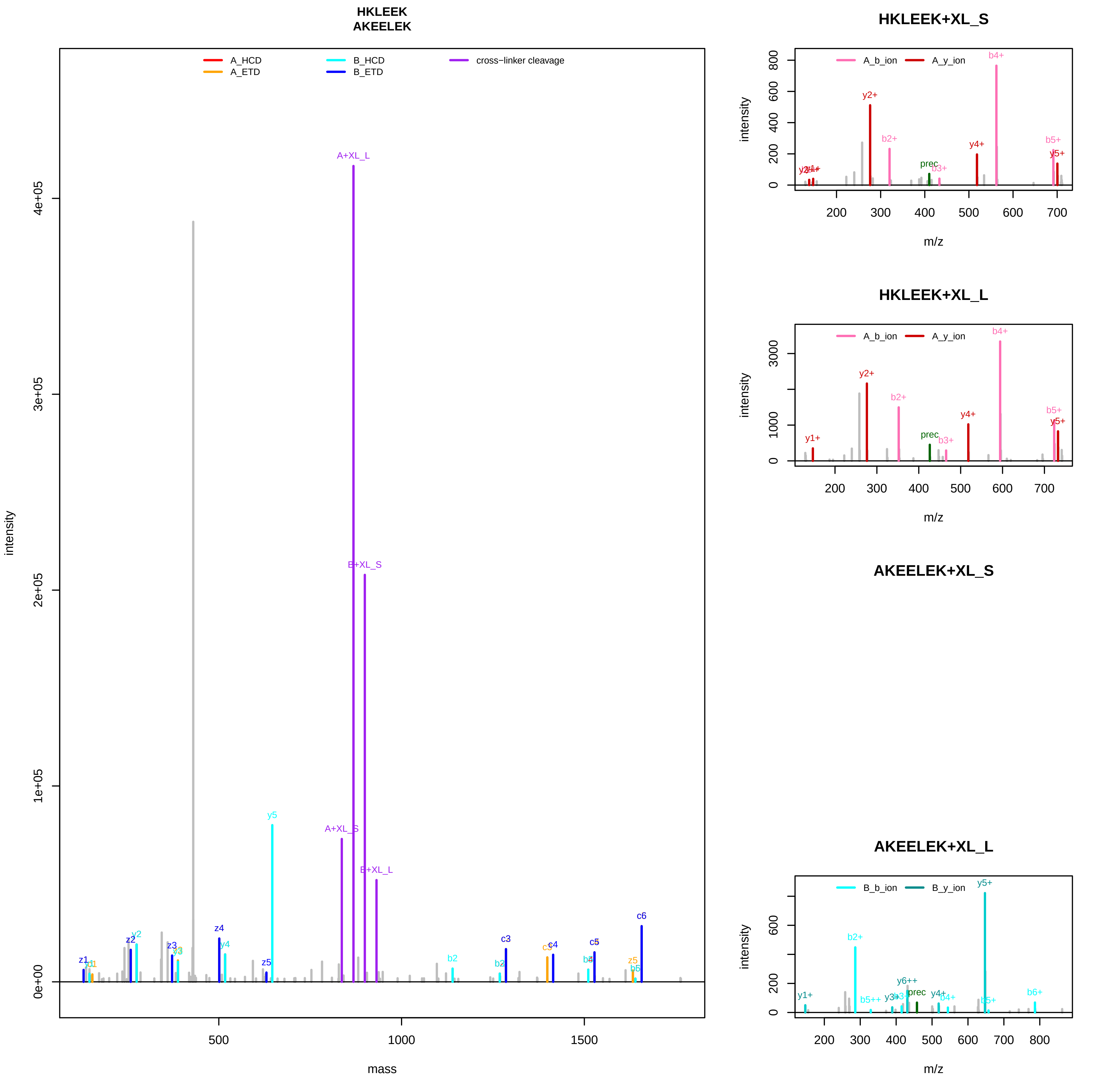
HKLEEK
AKEELEK
A_HCD
A_ETD
B_HCD
B_ETD
cross−linker cleavage
intensity
mass
4e+05
3e+05
2e+05
1e+05
0e+00
500
1000
1500
A+XL_L
B+XL_S
A+XL_S
B+XL_L
y5
c6
c3
c4
c5
z1
z2
y2
z3
z4
y4
z5
b2
b4
z5
b5
HKLEEK+XL_S
A_b_ion
A_y_ion
b4+
y2+
b2+
prec
b3+
y4+
b5+
y5+
m/z
HKLEEK+XL_L
y1+
AKEELEK+XL_S
AKEELEK+XL_L
B_b_ion
B_y_ion
y5+
b2+
y6++
b5++
prec
y4+
b4+
b6+
y1+
